# Dynamics of the Second Extracellular Loop Control Transducer Coupling of Peptide-Activated GPCRs

**DOI:** 10.3390/ijms241512197

**Published:** 2023-07-30

**Authors:** Marcel M. Wygas, Jeannette M. Laugwitz, Peter Schmidt, Matthias Elgeti, Anette Kaiser

**Affiliations:** 1 Faculty of Life Sciences, Institute of Biochemistry, Leipzig University, Brüderstr. 34, 04103 Leipzig, Germany; 2 Medical Faculty, Institute of Medical Physics and Biophysics, Leipzig University, Haertelstasse 16-18, 04107 Leipzig, Germany; 3 Medical Faculty, Institute for Drug Discovery, Leipzig University, Haertelstasse 16-18, 04107 Leipzig, Germany; 4 Medical Faculty, Department of Anesthesiology and Intensive Care, Leipzig University, Liebigstrasse 19, 04103 Leipzig, Germany

**Keywords:** peptide G-protein-coupled receptor (GPCR), extracellular loop, conserved disulfide, electron paramagnetic resonance (EPR), functional selectivity, arrestin

## Abstract

Many peptide-activated rhodopsin-like GPCRs share a β-hairpin folding motif in the extracellular loop 2 (ECL2), which interacts with the peptide ligand while at the same time being connected to transmembrane helix 3 (TM3) via a highly conserved disulfide bond. Currently, it remains unknown whether the coupling of the specifically shaped ECL2 to TM3 influences the activation of peptide-activated GPCRs. We investigated this possibility in a selection of peptide GPCRs with known structures. Most of the receptors with cysteine to alanine mutations folded like the respective wild-type and resided in the cell membrane, challenging pure folding stabilization by the disulfide bridge. G-protein signaling of the disulfide mutants was retained to a greater extent in secretin-like GPCRs than in rhodopsin-like GPCRs, while recruitment of arrestin was completely abolished in both groups, which may be linked to alterations in ligand residence time. We found a correlation between receptor activity of the neuropeptide Y_2_ receptor and alterations in ECL2 dynamics using engineered disulfide bridges or site-directed spin labeling and EPR spectroscopy. These data highlight the functional importance of the TM3-ECL2 link for the activation of specific signaling pathways in peptide-activated GPCRs, which might have implications for future drug discovery.

## 1. Introduction

G-protein-coupled receptors (GPCRs) form the largest group of transmembrane receptors and are activated by a variety of different ligand classes, including light, small molecules, ions, proteins, or peptides. They are involved in the majority of physiological processes, and thus, it is not surprising that they are targeted by over 30% of all FDA-approved drugs [1,2]. GPCRs share a common overall structure with seven transmembrane helices connected by three extracellular and three intracellular loops. However, they can be sub-classified into five phylogenetic families according to the GRAFS system [3], which display distinct sequence signatures in the transmembrane (TM) domain that correlate with differences in their activation mechanism. Previously, it had been assumed that GPCRs act as simple on/off switches. However, work of the last decade has made increasingly clear that they act as molecular modulators. A detailed look into the well-studied rhodopsin family [4,5,6], but also secretin-like [7,8] and frizzled receptors [9,10], reveals that GPCRs display high structural plasticity and molecular dynamics. This molecular flexibility not only allows for binding of different ligands but also for the occurrence of ligand-specific conformational changes upon receptor activation and, thus, signaling via multiple transducers from a single receptor [11,12]. 

Many efforts have been undertaken to identify common mechanisms of receptor activation in these families. This led to the discovery of a variety of macro- and microswitches, such as the DRY, NPxxY, or CWxP motifs, that are involved in the maintenance of the inactive state and the activation of rhodopsin-like GPCRs [13,14]. Instead, additional contacts, such as a glycine kink in TM7 or a leucine cluster in TM6, are characteristic for secretin-like GPCRs [13]. Despite the recent advances in the understanding of GPCR structural dynamics, it is noteworthy that most efforts were devoted to the transmembrane region, which generally displays a high sequence homology. In addition, the more diverse extracellular regions may also contribute to receptor activation. In this regard, an extracellular disulfide bond is conserved in many GPCRs (92% in rhodopsin-like GPCRs, 97% in secretin-like GPCRs) across all subclasses and chemical types of ligands [15]. Early on, it has been shown that this disulfide linkage facilitates GPCR folding and enhances ligand binding [16,17,18,19,20]. However, in light of the recent wealth of structural information, we should revisit whether this structural feature has a functional role during GPCR activation. In the present manuscript, we place particular emphasis on pathway-specific effects for different transducers and receptor subfamilies.

One particularly interesting instance are rhodopsin-like peptide-activated GPCRs. In this family, a conserved β-hairpin folding motif can be found in the extracellular loop 2 (ECL2) connected to transmembrane helix 3 via the highly conserved disulfide bond [21]. While in many systems it has been shown that the ECL2 is intimately involved in ligand binding [22,23,24,25,26,27,28,29], the influence of the TM3-ECL2 linkage via the conserved disulfide bond on ligand binding and pathway-specificity remains unclear. The observation of this common structural feature despite the phylogenetically only distant relation of different peptide-activated receptors (in α through δ-branch of the rhodopsin family, respectively) reinforces the idea of extracellular loops participating in domain coupling for GPCR activation [30,31]. We, therefore, hypothesized that the linkage to TM3 and the specifically folded ECL2 is part of a TM-ECL-spanning allosteric network involved in receptor activation that constitutes the bias switch which differently regulates downstream transducer coupling.

In this study, we investigated the effect of mutations of the conserved disulfide bond on expression, trafficking, ligand binding, and transducer coupling for a selection of rhodopsin-like peptide-activated GPCR as well as secretin-like receptors. The loss of the conserved disulfide bond was mostly tolerated during receptor folding. Using the neuropeptide Y_2_ receptor (Y_2_R) as a prototype, we show that specific ECL2 conformations and a level of flexibility are required for function. G protein signaling was generally affected to varying degrees, but arrestin recruitment was abolished in all tested variants, indicating that the conserved disulfide bond is required in a pathway-specific context. In addition, G-protein-coupling was significantly less affected in secretin-like GPCRs, suggesting that other structural elements, such as the N-terminal domain, may stabilize ligand binding and facilitate receptor activation.

## 2. Results

### 2.1. The Conserved Disulfide Controls ECL2 Dynamics and Function of the Y_2_R

**Figure 1:**
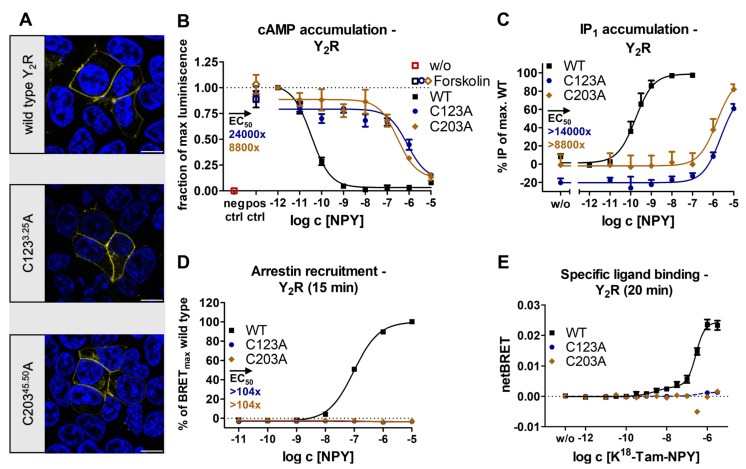
Expression and biological activity of Y_2_R variants with a mutation in the conserved disulfide bridge. (**A**) Live cell fluorescence microscopy shows a wild-type-like expression pattern of the disulfide bridge deficient variants. Pictures representative of two independent experiments, scale bar equals 10 µm. (**B**) Both Y_2_R variants showed a similar signaling profile in the native G_i/o_ pathway and remained fully activatable with 10 µM NPY. (**C**) Inositol monophosphate accumulation assay via a chimeric G_qi_ confirms the results of the cAMP accumulation assay with wild-type-like maximal activation. (**D**) Arrestin recruitment was not detectable to C123^3.25^A and C203^45.50^A variants in BRET-based assays, thus indicating an impairment of the arrestin pathway. (**E**) In NanoBRET-based ligand binding assays, specific binding of K^18^-TAMRA-NPY was not detectable for Y_2_R_C123^3.25^A and Y_2_R_C203^45.50^ variants, indicating loss of high affinity and low affinity binding states that are characteristic for the wild-type receptor. Data represent the normalized means ± SEM of *n* ≥ 5 (**B**), *n* ≥ 3 (**C**), *n* ≥ 2 (**D**), and *n* = 4 (**E**) independent experiments performed in technical triplicate.

NPY is the most abundant neuropeptide in the brain and is recognized by four GPCRs rendering it an excellent model system for functional regulation [32,33]. CryoEM has recently provided intriguing insights into peptide binding in the neuropeptide Y family [24]. Both structural snapshots and receptor mutagenesis suggest that the ECL2 represents an important epitope for peptide binding and selectivity and is essential for full activity of the receptor [24]. Given the high structural conservation of the two short antiparallel β-strands and their connection to TM3 via a disulfide bridge, we wondered whether the disulfide merely stabilizes the overall fold or if it may specifically contribute to receptor activation by enhancing the allosteric communication to the TM bundle. To probe this, we initially created two variants of the Y_2_R in which each of the cysteines of the conserved disulfide was individually exchanged for alanine. We expected similar outcomes if the effect was related to the loss of the disulfide, while environmental effects arising from positional mutagenesis could additionally affect a single variant. To our surprise, both Y_2_ receptor variants, Y_2_R_C123^3.25^A and Y_2_R_C203^45.50^A, folded well and were expressed at the cell membrane at levels comparable to the wild-type receptor (Figure 1A), enabling further investigations of receptor activity.

In the G_i_ pathway, the variants Y_2_R_C123^3.25^A and Y_2_R_C203^45.50^A substantially lost activity and displayed an EC_50_ that was shifted around four orders of magnitude compared to the wild-type Y_2_R, but full receptor activation occurred at 10 μM NPY concentration (Figure 1B). This result was confirmed using a chimeric G_qi_ protein [34] that redirects signaling from the native G_i_ to the G_q_ pathway and thus enables the detection of inositol phosphate accumulation (Figure 1C). In this assay setting, the EC_50_ of the disulfide mutants was shifted similarly for both mutants, which indicates that the severe reduction of receptor activity can be attributed to the lack of the disulfide linkage as such and not to the individual mutations. 

In order to test for possible pathway specificity, we examined the recruitment of arrestin3 to the Y_2_R and its variants via a BRET-based arrestin recruitment assay. We titrated the receptor-to-arrestin ratios to ensure donor saturation (see Methods for details). Under these conditions, NPY induced arrestin recruitment to wild-type Y_2_R with an EC_50_ of 96 nM, but no arrestin recruitment was detectable in the disulfide bridge deficient C123^3.25^A and C203^45.50^A variants at 10 µM NPY after 10 min, indicating an >100-fold impairment in the arrestin pathway (Figure 1D). Collectively, the signaling data indicated that the removal of the conserved disulfide bond profoundly disturbed the signaling of the Y_2_R, which was not caused by changes in membrane expression.

Next, we checked whether these signaling deficits were due to deficits in receptor activation or if ligand binding was also compromised. To this end, we performed NanoBRET-based ligand binding assays using a genetic fusion of a nanoliciferase to the N-terminus of the receptor and a tetramethylrhodamine (TAMRA)-labeled peptide ligand. To correct for unspecific binding, we subtracted the BRET values obtained in the presence of 30 µM of the unlabeled peptide NPY or the high-affinity Y_2_R antagonist JNJ-31020028 [23,35]. The wild-type receptor displayed a biphasic binding behavior showing a high-affinity state with an K_d_ of 2.3 nM, which represents the G-protein-bound active signaling state, as well as a low-affinity state with an K_d_ of 267 nM, which was attributed to transducer-free binding states [24]. In contrast to the observations in the wild-type, high- and low-affinity binding were abolished in the disulfide bridge deficient C123^3.25^A and C203^45.50^A variants, which indicated a severe loss of binding beyond levels that are detectable in our assay setting (Figure 1E).

**Figure 2:**
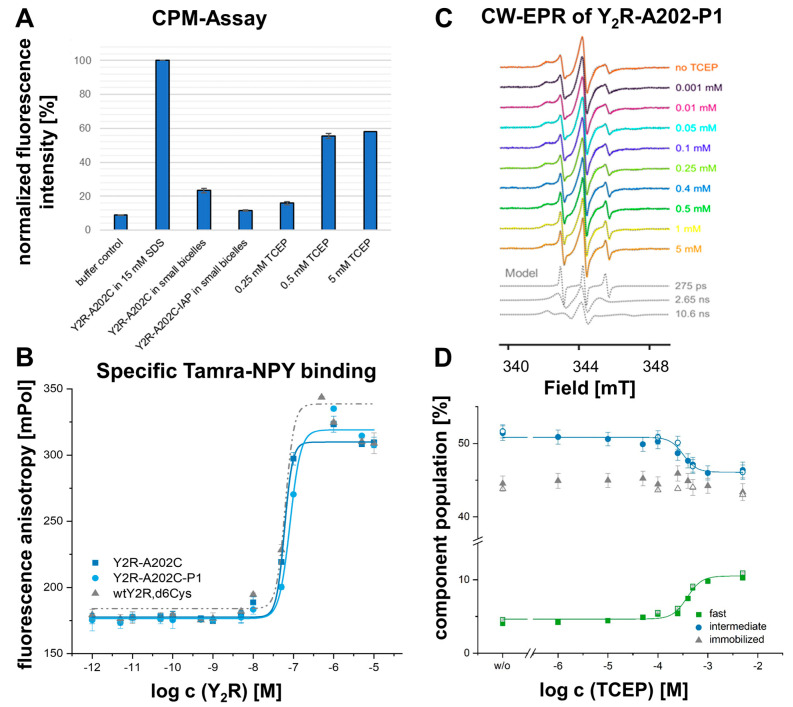
Conformational dynamics of ECL2 in Y_2_R. (**A**) Confirmation of disulfide bridge formation and IAP spin labeling in the in vitro Y_2_R preparations by CPM-Assay. Since this thiol-reactive fluorescent probe is essentially non-fluorescent until it reacts with free thiols, high fluorescence intensity values identify free cysteines. The unfolded Y_2_R-A202^45.49^C,Δ6Cys construct contains three accessible cysteines, whereas the receptor folded into small bicelles has only one free Cys, as shown by the decrease in fluorescence intensity. The fluorescence intensity of the IAP-labeled variant (no free Cys) is similar to the control sample and confirms the proper thioether reaction. Reduction of the native disulfide bond by TCEP again liberates two sulfhydryl groups, while the thioether-formed bond between C202 and IAP is kept stable. (**B**) Ligand binding capacity of Y_2_R-A202^45.49^C,Δ6Cys (dark blue), and A202^45.49^-P1 (light blue) in vitro preparations, all containing the intact disulfide, was tested within a fluorescence polarization assay using K^18^-TAMRA-NPY. Based on the saturation curve, K_d_-values of 61.4 ± 2.9 nM for Y_2_R-A202^45.49^C,Δ6Cys, and 81.8 ± 5.1 nM for A202^45.49^C-P1 were determined and are in the same nanomolar range as the K_d_-value of the wtY_2_RΔ6Cys with 63 ± 20 nM. (**C**) Room-temperature CW-EPR spectra of Y_2_R-A202^45.49^-P1 in the presence of varying amounts of reducing agent TCEP. All spectra represent a linear combination of three spectral components reflecting different dynamics (gray, bottom), which were determined by spectral fitting. (**D**) The titration of the reducing agent indicates a conformational equilibrium, which is modulated by the broken and intact disulfide bond, and which can be monitored by the population shift between components of fast and intermediate spin label dynamics, respectively. Data represent the normalized mean ± SEM of two independent experiments.

Based on this observation, we hypothesized that the removal of the structural constraint might have resulted in a changed conformation of the ECL2, leading to the observed effects on ligand binding and receptor signaling. We reasoned that the ECL2 might become highly flexible, rapidly fluctuating between multiple conformations; alternatively, the lack of covalent contacts with TM3 might lead to a collapse of the loop conformation and stable occlusion of the TM binding pocket. To answer this question, we employed continuous wave electron paramagnetic resonance spectroscopy (CW-EPR) and site-directed spin labeling [36] as a highly sensitive reporter of local structure and dynamics [37]. We chose position A202^45.49^ as the spin labeling site, which is located in the ECL2 directly next to the disulfide bridge. The background construct of the Y_2_R was depleted of solvent-accessible cysteines to avoid off-target labeling [38]. The final mutant construct (Y_2_R,Δ6Cys-A202^45.49^C) showed expression levels and G-protein activation similar to wild-type Y_2_R in HEK293 cells [39]. For CW-EPR analysis, Y_2_R was expressed in *E.coli*, purified, spin-labeled with iodoacetamide-proxyl (IAP) spin-label, and functionally reconstituted into phospholipid bicelles in vitro. We followed the Y_2_R folding process in vitro using a fluorescence-based assay employing the thiol-reactive fluorophore 7-diethylamino-3-(4′-maleimidylphenyl)-4-methylcoumarin (CPM). We confirmed the formation of the intramolecular disulfide bridge and successful spin labeling by alterations of CPM fluorescence (Figure 2A). Furthermore, we verified ligand binding for the spin-labeled receptor construct using established protocols (Figure 2B) [40,41].

We chose the IAP spin label as it forms a non-reducible thioether bond to the spin label side chain P1. This enabled CW-EPR experiments in the presence of reducing agents to open the conserved extracellular disulfide. For analysis of the CW-EPR line shapes, we chose a simple isotropic motional model, which proved adequate for the simulation of all CW-EPR spectra acquired. We also tested more complicated models, including anisotropic motion, broadening, and ordering potentials; however, with no significant improvement of the spectral fits. The room-temperature CW-EPR spectrum of Y_2_R_A202^45.49^-P1 (Figure 2C, no TCEP) is complex, caused by the superposition of at least two spectral components, each of which exhibit different spin label dynamics on the fast and intermediate nanosecond timescale. To gain a more quantitative picture of how the spin label dynamics and the underlying Y_2_R conformational states are modulated by the redox equilibrium of the disulfides, we performed a titration with the reducing agent tris(2-carboxyethyl)phosphine (TCEP). We detected a TCEP concentration-dependent change of the spectral line shape, namely an increase in intensity in the spectral regions characteristic of the most dynamic component (Figure 2C). CW-EPR line shape analysis suggests three components with different spin label dynamics, each characterized by a specific correlation time τ_c_ (Figure 2C). The fast and intermediate components appear to share a TCEP-dependent equilibrium (c^eq^ = 0.37 mM), and the immobilized component is constant across the entire TCEP titration (Figure 2D). 

Accordingly, reduction of the ECL2-TM3 disulfide is associated with faster spin label dynamics (fast component) and hence, increased mobility of ECL2. On the other hand, as the population of intact disulfide is reduced, the EPR component with intermediate spin label dynamics is decreased, suggesting that the fast and intermediate populations are in equilibrium. Importantly, a significant amount of fast-moving spin labels is present even in the absence of a reducing agent (~5%), which indicates the presence of the broken disulfide even under non-reducing conditions. Notably, we also observe a slow-moving immobilized component in all spectra. As its population is not affected even in the presence of a 5 mM reducing agent, we suspect that the slow component reflects nonfunctional receptors or aggregates formed during the refolding procedure. Taken together, our CW-EPR analysis of ECL2 dynamics in Y_2_R supports the hypothesis that a missing disulfide leads to increased loop dynamics, which is related to compromised ligand binding, G protein activation, and arrestin recruitment in cellular assays (cf. Figure 1).

**Figure 3:**
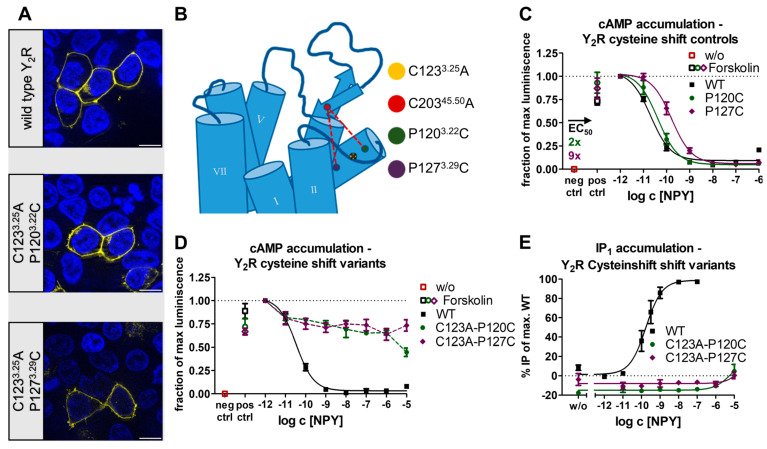
Expression and biological activity of Y_2_R cysteine shift variants. (**A**) Live cell fluorescence microscopy shows wild-type-like expression patterns of the disulfide-shifted variants. Pictures representative of two independent experiments, scale bar equals 10 µm. (**B**) Localization of the conserved cysteines C123^3.25^ and C203^45.50^ and the position of the alternative cysteines P120^3.22^C and P127^3.29^C. (**C**) Native G_i/o_ signaling was only mildly affected in the P120^3.22^C and P127^3.29^C control variants, thus allowing to test cysteine shift variants. (**D**) G-protein signaling was further reduced in the C123^3.25^A-P120^3.22^C and C123^3.25^A-P127^3.29^C cysteine shift variants. (**E**) Inositol monophosphate accumulation assays via a chimeric G_qi_ confirm the results of the cAMP assay and indicate an abolished G-protein signaling. Data represent the normalized means ± SEM of *n* ≥ 4 (**C**), *n* ≥ 3 (**D**), and *n* ≥ 2 (**E**) independent experiments performed in technical triplicate.

Following the finding of a highly flexible ECL2 in the absence of the disulfide constraint, we tested whether a re-fixation of the ECL2 to the TM3 via alternative disulfide bridges is sufficient to restore the signaling at the Y_2_R (Figure 3). For this, the cysteine at position C203^45.50^ was retained while C123^3.25^ was mutated to alanine, and the positions P120^3.22^ and P127^3.29^ were chosen as potential mutation sites as they were located on the same helix surface side as the native C3.25 but shifted by one helix winding up/downwards (P120^3.22^C/P127^3.29^C). To control for potential structural perturbations of mutating the proline residues in TM3 to cysteine, we introduced the P120^3.22^C and P129^3.29^C mutations in the presence of the native disulfide bridge (Figure 3C). While the P120^3.22^C variant showed nearly wild-type-like properties in the cAMP accumulation assay, the EC_50_ of the P127^3.29^C variant was moderately shifted by nine-fold compared to the wild-type receptor (Figure 3C). Next, P120^3.22^C or P127^3.29^C, respectively, were introduced into the C123^3.25^A_C203^45.50^ background to enable the formation of alternative disulfides. Compared to the single cysteine C123^3.25^A_C203^45.50^ base variant, in both constructs with alternative disulfides G-protein signaling was further reduced and appeared completely abolished at a 10 μM NPY concentration (Figure 3D,E). Given the very moderate effects of the single cysteine mutations P120^3.22^C or P127^3.29^C in the presence of the native disulfide, our findings suggest that the alternative disulfide bond has formed but fails to restore functionality and instead negatively affects activation.

**Figure 4:**
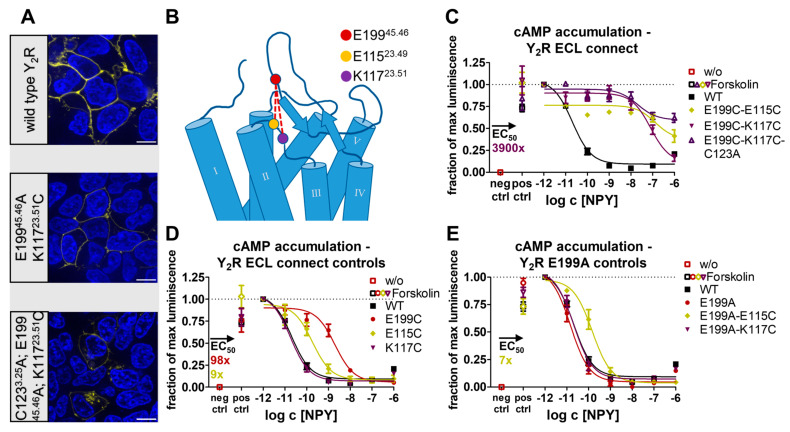
Expression and biological activity of Y_2_R ECL connect variants. (**A**) Live cell fluorescence microscopy shows the expression pattern of a selection of the disulfide shift (control) variants. While E199^45.46^A-K117^23.51^C was wild-type-like, the C123^3.25^A-E199^45.46^C-K117^23.51^C variant showed a higher degree of intracellular trapping. Pictures representative of two independent experiments, scale bar equals 10 µm. (**B**) Localization of the selected residues to force an additional disulfide between the ECL1 and the ECL2. (**C**) In the native G_i_ pathway, a severe loss of activity was detected for the E199^45.46^C-E115^23.49^C and E199^45.46^C-K117^23.51^C cysteine shift variants. When the E199^45.46^C-K117^23.51^C cysteine shift variant was combined with a C123^3.25^A mutation, the native G_i_ signaling was further reduced. (**D**,**E**) Cysteine shift control variants were tested for their activity in the native G_i_ pathway. E199^45.46^A appeared wild-type-like, while exchange to cysteine was less tolerated. K117^23.51^C and E199^45.46^A-K117^23.51^C were wild-type-like, while the G_i_ signaling of E115^23.51^C and E199^45.46^A-E115^23.51^C was mildly impaired, which indicated that both positions were suitable to form an additional disulfide. Data represent the normalized means ± SEM of *n* ≥ 2 (**C**), *n* ≥ 3 (**D**), and *n* ≥ 3 (**E**) independent experiments performed in technical triplicate.

These findings suggest that not only the presence but also the orientation of the conserved disulfide bridge are important for receptor function. We next chose to further limit the flexibility of the ECL2. For this, an additional constraint was introduced by connecting the ECL1 to the top of the β2-strand of the ECL2 in the presence of the conserved disulfide bond. Based on the cryo-EM structure of Y_2_R [24], we selected position E199^45.46^ in ECL2 as it appeared to be in a suitable range and orientation to ECL1. In ECL1, the positions E115^23.49^ and K117^23.51^ were chosen (Figure 4B). As a control, we exchanged E199^45.46^ for alanine and used this E199^45.46^A base variant to introduce E115^23.49^C and K117^23.51^C, respectively. In addition, the single cysteine variants E199^45.46^C, E115^23.49^C, and K117^23.51^C were created, and all constructs were tested in a cAMP accumulation assay (Figure 4C,D). While E199^45.46^A appeared wild-type-like, the introduction of a cysteine at this position resulted in a 98-fold shifted EC_50_ when compared to the wild-type. In both wild-type and E199^45.46^A background, K117^23.51^C displayed wild-type-like properties, underlining the suitability of this position to construct an additional disulfide bond. Similarly, the E115^23.49^C variants in the wild-type or E199^45.46^A background showed a seven to nine-fold shift in EC_50_, making this position also amenable to constructing a disulfide to E199^45.46^C. We next created the corresponding double cysteine (“ECL connect”) variants E199^45.46^C-E115^23.49^C and E199^45.46^C-K117^23.51^C that allow the formation of the additional disulfide. For both ECL connect variants, a similarly strong loss of activity was detected in the G_i/o_ pathway (Figure 4E). The E199^45.46^C-K117^23.51^C variant showed a 3900-fold shifted EC_50_, and the E199^45.46^C-E115^23.49^C appeared even slightly further right-shifted and did not reach saturation at 1 µM NPY, which was likely caused by additional position-specific effects previously detected in the E115^23.49^C control variants (compare Figure 4D). Overall, the reduced activity of both double disulfide constructs by far exceeded the combination of the single cysteine controls, suggesting that the additional disulfide has formed and that the resulting increased rigidity impairs receptor activation. 

Nevertheless, we wondered whether the tethering of ECL1 to ECL2 could be helpful in a context where the native disulfide is absent. To probe this, we created a triple mutant in which the conserved disulfide bridge was disrupted via C123^3.25^A while ECL1 was connected to ECL2 via E199^45.46^C and K117^23.51^C. This construct was even less active than the E199^45.46^C-K117^23.51^C ECL connect double variant (Figure 4E) and the disulfide bridge-deficient C123^3.25^A single variant (Figure 1B), which may be attributed to a moderately impaired membrane expression (Figure 4A). This reinforced the idea that the sole fixation of ECL2 to keep the ligand binding pocket open is not sufficient to ensure correct receptor function. Instead, a specific orientation and level of flexibility of ECL2 is required to enable specific conformations. 

### 2.2. Receptor- and Pathway-Specific Role of the Conserved Disulfide in Different Peptide-Activated Rhodopsin-like GPCRs

**Figure 5:**
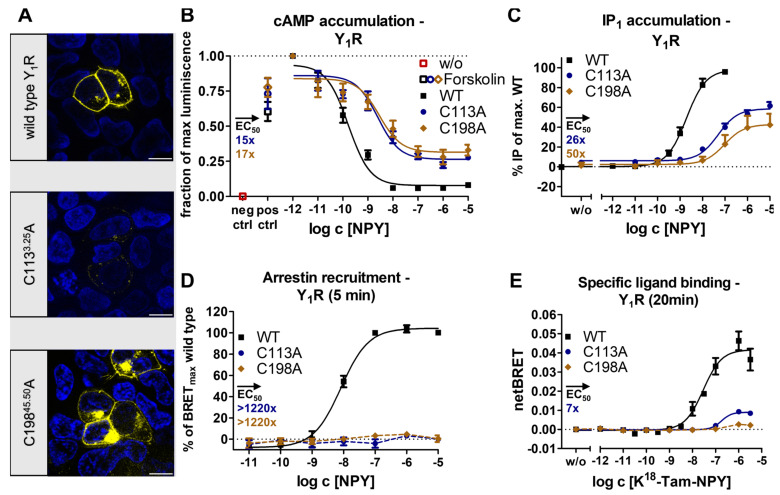
Expression and biological activity of Y_1_R variants with a mutation in the conserved disulfide bridge. (**A**) Live cell fluorescence microscopy shows a mildly reduced membrane expression of the disulfide bridge deficient variants. Pictures representative of two independent experiments, scale bar equals 10 µm. (**B**) Both Y_1_R variants showed a similar signaling profile in the native G_i/o_ pathway, indicating a mild impairment of the G-protein signaling. (**C**) Inositol monophosphate accumulation assays via a chimeric G-protein confirmed the results of cAMP accumulation (**D**) C113^3.25^A and C198^45.50^A variants of the Y_1_R did not recruit arrestin3 in BRET-based assays, thus indicating a severe impairment of the arrestin pathway. (**E**) In NanoBRET-based ligand binding assays, a severely reduced netBRET and a seven-fold shift in K_d_ were observed for K^18^-TAMRA-NPY binding to Y_1_R_C113^3.25^A, while no specific peptide binding was detectable for the Y_1_R_C198^45.50^A variant. Data represent the normalized means ± SEM of *n* ≥ 5 (**B**), *n* ≥ 3 (**C**), and *n* ≥ 2 (**D**,**E**) independent experiments performed in technical triplicate.

After investigating the role of the conserved disulfide for the signaling of the Y_2_R, we wondered whether the effects were universal to peptide-activated rhodopsin-like GPCRs. To test this, we turned to different receptors. First, we investigated possible differences within the neuropeptide Y receptor family and created disulfide bridge-deficient alanine variants for the Y_1_R. Both Y_1_R_C113^3.25^A and Y_1_R_C198^45.50^A variants were expressed in the plasma membrane; however, a higher degree of intracellular trapping was observed when compared to the wild-type (Figure 5A). In the G_i_ pathway, C113^3.25^A and C198^45.50^A showed a similar signaling profile with a 20–26% loss of E_max_ and a 15–17-fold shift in EC_50_, which was partially attributable to the reduced membrane expression of both variants (Figure 5B). In the inositol monophosphate accumulation assay using the chimeric G-protein, a 41% loss of E_max_ and 26-fold shifted EC_50_ was detected for C113^3.25^A, while C198^45.50^A appeared to be more strongly affected, showing a 57% loss of E_max_ and a 50-fold shift in EC_50_ (Figure 5C). This suggests position-specific effects for the C198^45.50^A Y_1_R variant. Nevertheless, both assay systems indicated that the loss of the conserved disulfide bond only mildly affected the native G_i_ signaling, which contrasted with the observed severe impairment of the G_i_ signaling in the Y_2_R. 

Following this, we monitored the recruitment of arrestin3 to the Y_1_R and the disulfide-deficient variants. Interestingly, no arrestin recruitment was detected in the disulfide deficient variants at 10 µM NPY, even though the Y_1_R is generally known to have a high affinity to arrestin3 [42] and showed half-maximal recruitment at 8 nM NPY. Accordingly, loss of the disulfide resulted in a >1220-fold loss in functional affinity for this pathway (Figure 5D).

Based on these findings, we concluded that loss of the conserved disulfide bridge leads to G-protein bias in the Y_1_R. Since this may be explained by changes in the ligand binding pocket, we used the NanoBRET-based ligand binding assay to monitor affinity, but also (changes in) the binding orientation of the Tam-labeled NPY to the receptor variants. Interestingly, the netBRET signal of specific binding of the alanine variants was strongly reduced but at least saturable for C113^3.25^A with a seven-fold shift in K_d_ compared to the wild-type Y_1_R. The shift in binding affinity correlates well with the observed reduction of functional affinity in the G-protein pathway. The differences in the BRET window indicate a different binding orientation of the peptide at the C113^3.25^A variant of Y_1_R. Due to the very low signal window for the Y_1_R_C198^45.50^A construct, it remains unclear whether specific binding is precluded or occurs with very unfavorable BRET geometry that cannot be reliably detected, even though the functionality in the G-protein pathway rather argues for the latter scenario.

Together, these data suggest that the presence of the conserved disulfide bond is neither required for folding nor G_i_ signaling of Y_1_R. In contrast, the disulfide was required for arrestin3 recruitment by Y_1_R. This may arise from changes in the orientation of the peptide in the binding pocket upon removal of the conserved disulfide bond.

**Figure 6:**
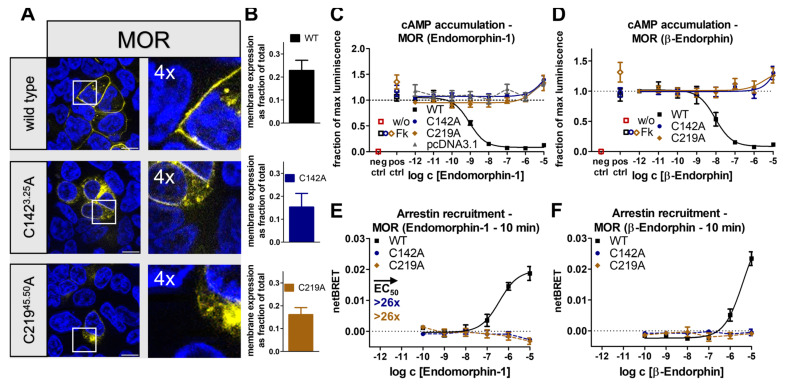
Expression and biological activity of MOR variants with a mutation in the conserved disulfide bridge. (**A**) Live cell fluorescence microscopy in HEK293 cells shows a reduced yet detectable membrane integration of the disulfide bridge deficient variants. Pictures representative of two independent experiments, scale bar equals 10 µm. (**B**) Cell surface ELISA verifies membrane expression of wild-type MOR and the disulfide-deficient variants. (**C**,**D**) cAMP reporter gene assays indicate a complete loss of the native G_i_ signaling in the C142^3.25^A and C219^45.50^A variants stimulated by the short endogenous agonist endomorphin-1 (**C**) or the long endogenous agonist β-endorphin (**D**). An unspecific concentration-dependent increase in the cAMP level was detected for both variants and ligands. (**E**,**F**) Arrestin recruitment to the C142^3.25^A and C219^45.50^A variants was not detectable in BRET-based assays, irrespective of the ligand used. Data represent the normalized means ± SEM of *n* = 3 (**B**), *n* ≥ 4 (**C**), *n* ≥ 4 (**D**), *n* ≥ 2 (**E**), and *n* ≥ 2 (**F**) independent experiments performed in technical triplicate.

In addition to the receptors of the Y-receptor family, we chose to study two other peptide-activated receptors, the µ-type opioid receptor (MOR) and the angiotensin II type 1 receptor (AT_1_R). Analogous to the workflow with NPY receptors, the conserved disulfide bond was removed via single alanine variants, and the expression of the receptor variants was first monitored by fluorescence microscopy. In contrast to the receptor variants of the Y receptor family, the membrane integration of the generated MOR variants MOR_C142^3.25^A and MOR_C219^45.50^A was severely impaired, which resulted in a high proportion of intracellularly located receptors (Figure 6A). We confirmed cell-surface expression by ELISA (Figure 6F) in agreement with a previous study [43], which allowed for functional characterization. As the MOR is known to be targeted by several endogenous peptides, we chose to use peptides of different lengths in combination with the receptor variants and used the short 4-mer endomorphin-1 and the longer 31-mer peptide β-endorphin. While wild-type MOR was activated by endomorphin-1 and β-endorphin with EC_50_ values of 0.8 nM and 9.2 nM in the G_i_ pathway, respectively, G_i_ signaling was lost in both disulfide deficient variants when stimulated by either ligand (Figure 6B,C). Interestingly, an increase in cAMP levels was detected at high concentrations of endomorphin-1 and β-endorphin in both wild-type MOR and its cysteine mutants. For wild-type MOR, such a G_i_-to-G_s_ switch has previously been proposed [44,45,46]. However, a similar response is also seen in untransfected cells and is, therefore, likely unspecific. We further wondered whether MOR variants were still able to recruit arrestin. For wild-type MOR, arrestin3 recruitment was measured for both ligands with EC_50_ of 375 nM for endomorphin-1 and >3000 nM for β-endorphin, respectively. Interestingly, despite wild-type stimulation with 10 µM β-endorphin showing no saturation, a higher BRET_total_ was detected when compared to endomorphin-1, which suggested differences in the orientation of the receptor-arrestin complexes. In contrast, arrestin recruitment was not observed for the MOR_C142^3.25^A and MOR_C219^45.50^A variants (Figure 6D,E) using 10 µM with either peptide ligand. Combined, the data indicate that the removal of the conserved disulfide bond severely impaired the signaling of the MOR.

**Figure 7:**
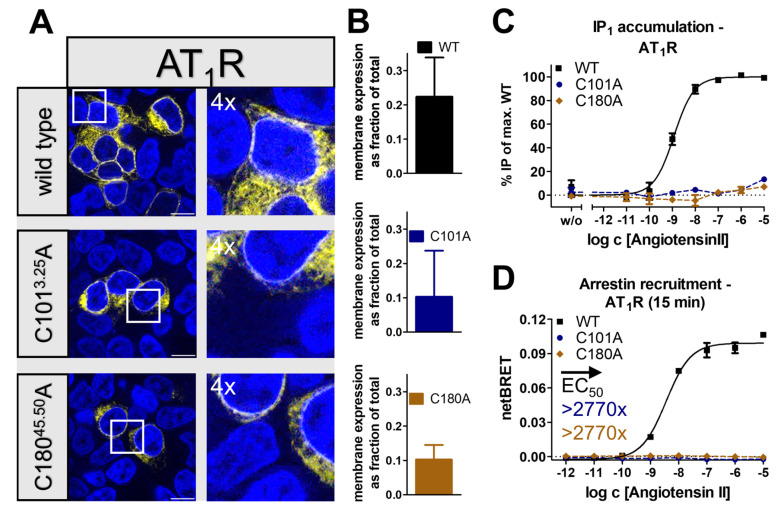
Expression and biological activity of AT_1_R variants with a mutation in the conserved disulfide bridge. (**A**) Live cell fluorescence microscopy in HEK293 cells showed a poor membrane expression of wild-type AT_1_R and disulfide bridge deficient variants. Pictures representative of two independent experiments, scale bar equals 10 µm. (**B**) Cell surface ELISA confirms membrane expression of wild-type AT_1_R and disulfide-deficient variants. (**C**) Inositol monophosphate accumulation assays indicate a nearly complete loss of the native G_q_ signaling. (**D**) Arrestin3 was not recruited to the C101^3.25^A and C180^45.50^A variants in BRET-based assays, thus indicating a severe impairment of the arrestin pathway. Data represent the normalized means ± SEM of *n* ≥ 2 (**B**), *n* ≥ 2 (**C**), and *n* ≥ 2 (**D**) independent experiments performed in technical triplicate.

In contrast to the other selected receptors, the AT_1_R was the only receptor in which the wild-type already showed only fractional membrane expression in transfected HEK293 and a high content of intracellularly located receptors (Figure 7A). A similar picture was also observed for the AT_1_R_C101^3.25^A and AT_1_R_C180^45.50^A variants; however, membrane integration was still detectable. We corroborated these findings with a cell surface ELISA (Figure 7D). In terms of G_q_ signaling, AT_1_R_C101^3.25^A, and AT_1_R_C180^45.50^A were hardly activated by the endogenous ligand angiotensin II (Figure 7B) compared to the wild-type that was activated with an EC_50_ of 1.2 nM. Severe loss of function was also detected in the arrestin pathway. Arrestin3 was recruited by wild-type AT_1_R with an EC_50_ of 3.6 nM but not to either disulfide bond deficient variant, indicating an >2770-fold impaired arrestin recruitment (Figure 7C). Taken together, the observations showed that the disruption of the conserved disulfide bridge receptor severely influenced receptor folding, arrestin recruitment, and G_q_ signaling, leading to an essentially inactive receptor.

### 2.3. The Conserved Disulfide Bond Is Not Required for G-Protein Activation of Secretin-like GPCRs

**Figure 8:**
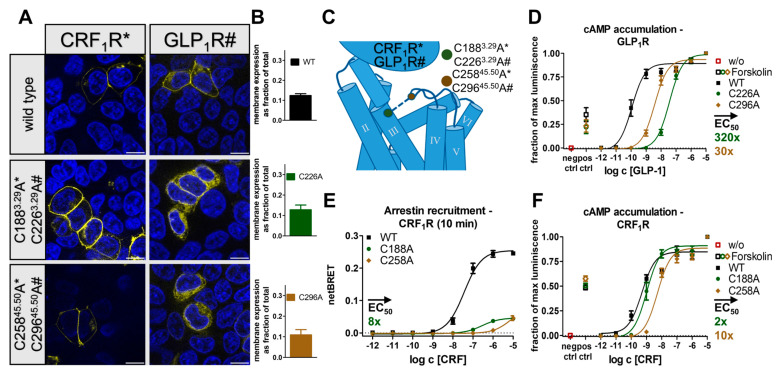
Expression and biological activity of CRF_1_R and GLP_1_R variants with a mutation in the conserved disulfide bridge. (**A**) Live cell fluorescence microscopy in HEK293 cells shows a wild-type-like expression of the CRF_1_R variants, while the GLP_1_R variants show a higher degree of intracellular trapping. Pictures representative of two independent experiments, scale bar equals 10 µm. (**B**) Cell surface ELISA to verify membrane expression of the wild-type GLP_1_R and the disulfide deficient variants. (**C**) Schematic representation of residues that are involved in the formation of the conserved disulfide. (**D**) GLP_1_R variants were moderately less potent in the G_s_ pathway. (**E**) BRET-based arrestin recruitment assays reveal a severely reduced arrestin recruitment to the CRF_1_R variants. (**F**) In the native G_s_ pathway, CRF_1_R variants showed a wild-type-like (CRF188^3.29^A) or mildly impaired (CRF258^45.45^A) signaling. Data represent the normalized means ± SEM of *n* = 7 (**B**), *n* = 4 (**D**), *n* ≥ 2 (**E**), and *n*= 4 (**F**) independent experiments performed in technical triplicate.

Contrary to peptide-activated rhodopsin-like GPCRs, secretin-like GPCRs typically do not display a β-hairpin motif in the ECL2, which is accompanied by an overall shorter length of the ECL2 [31,47,48]. Nonetheless, the disulfide bridge between the ECL2 and TM3 is highly conserved in this subfamily as well [15], and the conserved cysteines are denoted as 3.29 and 45.50 in the Wootten numbering scheme [49]. To probe to which extent this disulfide is functionally required in secretin-like GPCRs similar to the rhodopsin-like peptide-activated subtypes, we selected two members of the secretin-like family; the corticotropin-releasing factor receptor 1 (CRF_1_R) and the glucagon-like peptide 1 receptor (GLP_1_R). Analogous to our approach for the rhodopsin-like GPCRs, two variants were created for each receptor in which the cysteines involved in the formation of the conserved disulfide bond were individually exchanged to alanine (Figure 8B). While the expression of the CRF_1_R_C188^3.29^A and CRF_1_R_C258^45.50^A variants appeared to be wild-type-like, both variants of the GLP_1_R showed reduced membrane integration with an increase in the amount of intracellularly located receptor (Figure 8A). We confirmed cell surface expression by cell surface ELISA (Figure 8F). cAMP accumulation assays were performed to monitor the signaling of the variants in their native G_s_ pathway (Figure 8C,E). In the GLP_1_R variants, the E_max_ remained unaffected, and the EC_50_ of the GLP_1_R_C296^45.50^A variant was moderately right-shifted by 33-fold. The functional effects for GLP_1_R_C226^3.29^A were stronger and showed a 322-fold shift in EC_50_, in agreement with previously described position-specific effects of the free cysteine at position C226^3.29^A in GLP_1_R [50]. Similarly, for the CRF_1_R, variant CRF_1_R_C188^3.29^A was nearly wild-type-like with no changes in E_max_ and only a two-fold shift in the EC_50_, while CRF_1_R_C258^45.50^A was 10-fold right shifted when compared to the wild-type. Nonetheless, since the *minimal* functional change of either of the alanine mutants reflects the importance of the disulfide bridge, these effects were overall very mild for G_s_ activation of GLP_1_R and CRF_1_R despite the partially reduced membrane integration of the GLP_1_R variants. We, therefore, concluded that the conserved disulfide bond is not required for the folding and G-protein signaling of these secretin-like GPCRs. 

While some form of G-protein signaling was also detectable in the disulfide bridge-deficient variants of the selected rhodopsin-like GPCRs, recruitment of arrestin3 was always lost under the given experimental conditions. We therefore tested whether the presence of the conserved disulfide bond was also a prerequisite for arrestin recruitment to peptide-activated secretin-like GPCRs. For this, we measured arrestin3 recruitment at the CRF_1_R wild-type and the CRF_1_R_C188^3.29^A and CRF_1_R_C258^45.50^A variants by BRET. The wild-type CRF_1_R recruited arrestin3 with an EC_50_ of 37 nM, while the CRF_1_R_C188^C3.29^A variant showed an eight-fold shift in EC_50_ as well as an 80% reduced BRET_max_ (Figure 8D). Arrestin recruitment to the CRF_1_R_C258^45.50^A variant was even further blunted and did not reach saturation up to 10 µM CRF. Together, the data suggest that the presence of the conserved disulfide bond is required for the efficient recruitment of arrestin3 to secretin-like GPCRs, even in instances where G-protein activation is wild-type-like in the absence of the disulfide. 

## 3. Discussion

The extracellular disulfide has generally been implicated in maintaining the integrity of the GPCR fold [21]. Additionally, it connects the β-hairpin folding motif conserved in the ECL2 of peptide-activated rhodopsin-like GPCRs to the TM3, which has mainly been considered to keep the binding pocket open [48]. Our data show that the conserved disulfide bond is not generally required for the structural integrity of peptide-activated GPCRs, as receptor variants lacking the disulfide are still membrane-expressed. In regards to signaling, the effects are very specific to each receptor and signaling pathway. G-protein activation is affected differently, but a common effect is seen for the recruitment of arrestin3, which is severely reduced and in most cases completely undetectable. CW-EPR experiments and the introduction of additional disulfides into the Y_2_R further indicate that a specific level of flexibility of the ECL2 is required for function.

We have initially used the human Y_1_ and Y_2_ receptors as prototypes to interrogate the functional contribution of the extracellular disulfide for the activation of G-proteins versus arrestin recruitment. Both receptor subtypes tolerated the loss of the disulfide very well for folding (compare Figure 1 and Figure 5), enabling unambiguous functional interpretations. While the Y_2_R variants showed over 8000-fold reduced potency to activate G-proteins, G-protein activation of the corresponding Y_1_R variants was only moderately affected with ~20-fold shifted EC_50_. Interestingly, however, recruitment of arrestin3 to both mutated Y_1_R and Y_2_R was completely abrogated, equivalent to >1220-fold and >104-fold-reduced functional potencies. We note that there is hardly any receptor reserve in our G-protein activation assay setup [22]; therefore, the assays for G-protein activation and arrestin-recruitment have similar sensitivity. While at the Y_2_R, the potency to recruit arrestin3 was already very low at the wild-type receptor, precluding any conclusions on potential receptor bias, cysteine mutants of the Y_1_R were biased towards G-protein activation. Y_1_ and Y_2_ receptor subtypes are prototypic model systems for different modes of arrestin-recruitment, termed class A and class B arrestin-recruiting receptors [42,51,52,53]. Class A arrestin-recruiting receptors such as the Y_1_R co-internalize with arrestin bound and have a tight interaction of their phosphorylated C-termini with arrestin, enabling stable interactions in a ‘tail’-configuration that does not require contacts of the TM domain with arrestin finger- or middle loops [42,51]. Class B type arrestin-recruiting receptors such as the Y_2_R display arrestin interactions only at the plasma membrane, and the protein complex involves contacts of the receptor TM core and phosphorylated C-terminal tail [42,51]. Both interaction types seem to be strongly destabilized by mutation of the disulfide bridge, possibly already at the level of receptor phosphorylation by GRKs or ligand residence time. Arrestin recruitment to GPCRs is a slow multistep process. We suggest that the disulfide increases ligand residence time, which is essential for efficient GPCR phosphorylation and arrestin binding. Interestingly, a reduction in ligand residence time in LSD-stimulated 5-HT_2A_R and 5-HT_2B_R variants bearing a mutation in the ECL2 was previously shown to reduce the potency of β-arrestin-2 recruitment but not of the G_q_-mediated signaling, which supports this hypothesis [54]. Similarly, cysteine mutants of AT_1_R and MOR also showed no detectable interaction with arrestin, and even the secretin-family CRF_1_R that has very mild effects on G-protein activation has drastically reduced arrestin recruitment (see below).

G-protein activation of the diverse GPCRs tested was affected much differently. The effects were not correlated with different types of G-protein, as G_i_ activation was moderately (Y_1_R), very strongly (Y_2_R), or completely (MOR) abrogated, respectively. G_q_ coupling of the AT_1_R mutants was completely absent. Therefore, the specific effects are likely related to the individual architecture of the respective binding pockets. 

The AT_1_R was essentially inactive upon loss of the conserved disulfide. In this receptor, the conserved β-hairpin structure is extended by another β-strand, which is contributed by the distal N-terminus. Interestingly, a second extracellular disulfide links the membrane-proximal part of the NT with ECL3. This structure limits the accessibility of and leads to a narrower binding pocket [27,28]. It is likely that the loss of the structural constraint increases the flexibility of the β2-strand, allowing it to move into the binding pocket and, thus, occlude ligand binding to the AT_1_R.

Next, we selected the µ-type opioid receptor (MOR), which is activated by a variety of structurally diverse ligands [25,26]. No specific activation was detectable in the disulfide-deficient variants stimulated by the endogenous peptide β-endorphin (31mer) bearing the common C-terminal YGGF opioid motif as well as the short peptide endomorphin-1 (4mer) consisting of the modified YPWF motif. While the two ligands appear heterologous, structural data reveal that both stabilize similar active structures [26]. The ligand binding pocket of the MOR appears to be quite large. In contrast to the short peptide endomorphin-1, whose interactions are limited to the deeper binding pocket, it was shown that β-endorphin additionally interacts with the ECL2, which in the MOR was proposed to function as a selectivity filter for different opioid peptides, and the extracellular ends of TM1 and TM2 [26]. These additional contacts to the ECL2 are not sufficient to rescue the canonical G-protein signaling. Instead, loss of the extracellular disulfide seems to loosen the hydrophobic binding pocket between ECL1 and TM3 that accommodates the opioid “message” sequence YGGF/YPWF or may affect ligand residence time.

While the MOR was used as a prototype for a receptor activated by structurally different peptides, we also looked into the Y_1_R and Y_2_R, which are both activated by the same endogenous ligand NPY and hence have more similar steric requirements in the binding pocket. G-protein signaling was markedly less affected in Y_1_R than in Y_2_R. We speculate that additional interactions stabilize NPY binding to the Y_1_R in the absence of the conserved disulfide and ameliorate the effects. Indeed, several differences in the ligand binding mode of NPY to the Y receptors can be found [22,23,24]. Most notably, the Y_1_R possesses an additional binding pocket for the N-terminus of NPY, which contributes to ligand binding and signaling [24]. In the active Y_1_R, the positions of the extracellular ends of the TM helices and EC loops are more similar to the inactive state. In the NPY-bound Y_2_R structure, the extracellular ends of TM2 and TM6 contract towards the agonist in a pincer-like movement compared to the inactive snapshot [23]. The ECL2 strongly engages in interactions with a hydrophobic patch in the NPY helix, which is crucial for receptor binding and the correct orientation of the C-terminal residues involved in receptor activation [24]. It appears reasonable that the increased flexibility of the ECL2 induced by the removal of the conserved disulfide would destabilize essential contacts to the peptide and hence the contraction of the extracellular parts of the receptor. While this effect would certainly take place in both receptors, the overall reduced number of interactions in the Y_2_R renders the receptor more prone to changes in the main interaction patch. 

The ECL2 of Y_1_R and Y_2_R are amongst the most mobile components of the receptor, as judged by the low local resolutions in the cryo-EM structures [24]. We turned to CW-EPR with purified receptors that carry a site-specific label in ECL2 to investigate changes in loop flexibility in more detail. Surprisingly, the EPR signal contained signatures of fast and intermediate mobility already in the basal state, suggesting that there is an equilibrium of an oxidized and reduced disulfide state that might both contribute to the specific inherent functional profile of Y_2_R. This chemical reduction by adding TCEP increased the fast mobility component, providing a molecular basis for the interpretation of our functional data. Reduction of the conserved disulfide bridge did not lead to a collapse of the binding pocket but rather indicated an increase in ECL2 flexibility. This data is in accordance with early FTIR spectroscopic results on rhodopsin activation that suggested a transient cleavage of the extracellular disulfide occurring in one of the activation intermediates [55] and implies that in this more flexible ECL2 conformation peptide-receptor contacts are weakened. 

**Figure 9:**
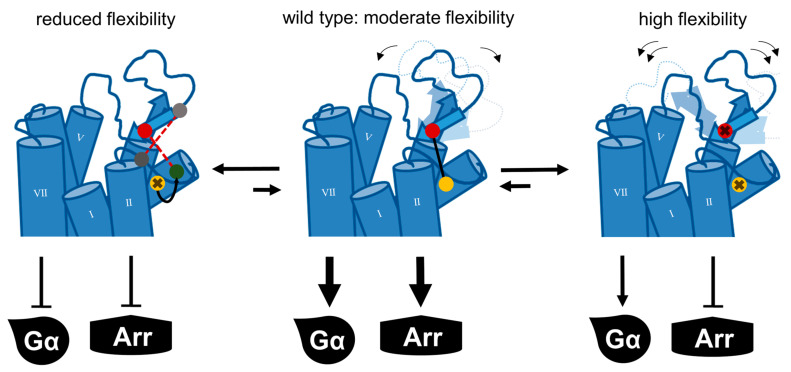
Fine-tuned flexibility of the ECL2 is important for the function of the Y_2_R. In the wild-type receptor (**middle**), which is fully capable of recruiting arrestin3 and activating the Gi pathway, the ECL2 is constrained via the conserved disulfide bond and displays moderate flexibility. When the conserved disulfide is lost (**right**), the flexibility of the ECL2 is increased. G-protein signaling is severely impaired yet still detectable, while arrestin recruitment is completely abolished. In the opposite scenario (**left**), the ECL2 flexibility is reduced by introducing additional disulfide bridges or a shift in the positioning of the conserved disulfide bond. In this case, arrestin recruitment and G-protein signaling are nearly completely lost.

Structural snapshots obtained via cryo-EM showed that the ECL2 may be flexible enough to interact with ECL1 [24]. Thus, we introduced additional cysteines into the ECL2 and ECL1 to introduce an additional disulfide. The accompanying reduction in flexibility severely impaired receptor function (compare Figure 4). Moreover, shifting the connection to TM3 by one helix winding from C3.25 to 3.22 or 3.29 rendered the receptor completely inactive. Thus, our results indicate that the conformational dynamics of ECL2 are tightly regulated by the exact positioning of the disulfide, which is essential for the activity of Y_2_R (Figure 9). 

Contrasting to the specific but often severe functional impairments of rhodopsin-like receptors resulting from removing the conserved disulfide, the effects on G-protein signaling remain mild for the tested secretin-like GPCRs CRF_1_R and GLP_1_R. Surprisingly, arrestin recruitment was still severely impaired, with an 80% reduced BRET window in the CRF_1_R variants. Based on this finding, we speculate that specific structural features of secretin-like GPCRs stabilize ligand binding and G-protein activation but are not sufficient to trigger arrestin interactions. In contrast to rhodopsin-like receptors, in secretin-like GPCRs, a larger and well-ordered NT domain strongly stabilizes peptide binding [3,56]. This seems to be a specific adaptation to peptide ligands [3,56], while the rhodopsin family displays a variety of ligand classes with varying sizes. In turn, the ECL2 of secretin-like receptors is shorter and lacks the β-hairpin folding motif [57]. While rhodopsin-like and secretin-like GPCRs form separate phylogenetic clusters [3], it is hypothesized that both classes derive from a common ancestral peptide receptor that already contains the β-hairpin motif [58]. This raises the question of whether the original function of the specifically folded ECL2 was at least partially transferred to the newly occurring N-terminal domain, thus lowering the evolutionary pressure to retain previously conserved structural elements. Nonetheless, the ECL2 of secretin-like receptors is still involved in peptide binding [50,59,60,61,62,63], and the TM3-ECL2 linking disulfide bond remains highly conserved [15]. While not essential for G-protein activation, the ECL2 might also be involved in fine-tuning ligand residence time. In the current model, ligand binding to secretin-like GPCRs is composed of a two-step-based mechanism [62,64,65,66,67]. First, the C-terminal fragment of the ligand binds to the N-terminal domain of the receptor which triggers structural rearrangements inside the ligand. Next, the N-terminal fragment binds the orthosteric binding pocket of the receptor, which leads to receptor activation. In this scenario, we hypothesize that the disruption of the conserved disulfide still allows for the normal binding procedure. In the fully bound state, however, the increased flexibility of the ECL2 destabilizes interactions of the TM domain to the N-terminal part of the ligand, which may reduce ligand residence time. The ligand might then revert to a half-bound state in which its C-terminal ligand fragment remains bound to the N-terminal extracellular domain of the receptor while the peptide’s N terminus partly dissociates from its ‘final’ binding position, which destabilizes the active conformation. In contrast to rhodopsin-like receptors in which an immediate full dissociation of the ligand-receptor-complex is expected after the loss of the conserved disulfide, the two-step mechanism of secretin-like GPCR would allow for a fast re-association of the active complex, explaining the milder effect on G-protein coupling, while recruitment of arrestin requires a long-lived, fully engaged complex.

## 4. Conclusions

The present study demonstrates that the function of the conserved disulfide bond in peptide-activated GPCRs exceeds an involvement in GPCR folding. Its removal in peptide-activated receptors leads to a receptor-specific impairment of G-protein signaling, while arrestin recruitment is completely abolished. Experiments with the Y_2_R further show that the disulfide bond maintains a specific level of flexibility of the ECL2. We found an equilibrium of oxidized and reduced disulfide bonds for Y_2_R, with the cleaved disulfide conformation exhibiting increased ECL2 dynamics. We hypothesize that the disulfide bridge is important to increase ligand residence time. The cleavage of the disulfide leads to a shift of the conformational equilibrium manifesting in altered ligand- and transducer binding properties, which eventually leads to impairment of G-protein signaling and quantitative depletion of arrestin recruitment.

## 5. Methods

### 5.1. Generation of Plasmids

We used a genetic fusion of the receptors and variants of the enhanced green fluorescent protein (eGFP, eYFP) for transient transfection in all assay systems. For cAMP reporter gene assays, inositol monophosphate accumulation assays, BRET-based arrestin recruitment assays, fluorescence microscopy, and cell surface ELISA HA-Y_2_R-eYFP_N1, Y_1_R-eYFP_N1, AT_1_R-eYFP_N1, MOR-eYFP_pVitro2, GLP_1_R-eGFP_pcDNA3.1 constructs were used, which all carried the canonical receptor isoform. For the FLAG-CRF_1_R-eGFP_N1 receptor, the R2 isoform (P34998-2) was used. For nanoBRET-based binding assays, we devised the previously described N-terminally nanoluciferase-fused Nluc-Y_2_R-eYFP_N1 and Nluc-Y_1_R-eYFP_N1 constructs [24]. Receptor variants bearing the required point mutations were generated by site-directed mutagenesis using partially overlapping primers as described [68]. The plasmids were amplified in E. coli DH5α, purified using the ZymoPure II Kit (Zymo Research, Irvine, CA, USA), and all sequences were verified by Sanger sequencing.

### 5.2. Peptide Synthesis

Peptide synthesis was performed using the fluorenylmethoxycarbonyl (Fmoc)/*tert*-Butyl (*t*Bu) strategy in 15 µmol scale in a Syro II robot (Multisyntech, Witten, Germany) as described previously [69]. Briefly, for the generation of C-terminally amidated peptides, Rink amide or TGRam resins were used. For peptides containing C-terminal free acids, pre-loaded Wang resins were elongated. The peptide sequence was built using repeated cycles of Fmoc-deprotection with 40%/20% piperidine in DMF for 3 min and 10 min and double coupling of 8 eq. Fmoc-amino acid with 8 eq. Oxyma (ethyl 2-cyano-2-(hydroxyimino)acetate) for 45 min each. The 5/6-tetramethylrhodamine (TAMRA) fluorophore was coupled manually via the free acid at an orthogonally protected Lys(Dde) using 2 eq. TAMRA, 1.9 eq. HATU (1-[Bis(dimethylamino)methylene]-1H-1,2,3-triazolo[4,5-b]pyridinium 3-oxide hexafluorophosphate, hexafluorophosphate azabenzotriazole tetramethyl uronium) and 2 eq. diisopropylethylamine in DMF overnight as described [23]. Resins and protected amino acids were purchased from Iris Biotech (Marktredwitz, Germany). Peptide purification and analytics were performed by preparative/analytical RP-HPLC (Shimadzu/VWR) using gradients of acetonitrile in water at pH 2 as eluents on Aeris Peptide 5u XB-C18, Jupiter 4u Proteo 90 Å (C12), or Aeris Peptide 3,6 n XB-C18 columns (Phenomenex, Torrance, CA, USA). The purity of all peptides was > 95%. In addition, MALDI (Bruker, Billerica, MA, USA) Ultraflex III MALDI TOF/TOF) and ESI-Orbitrap-based mass spectrometry (Bruker, Billerica, MA, USA) was used to confirm peptide identities.

### 5.3. Cell Culture

All cell-based experiments were performed in HEK293 cells (human embryonic kidney, DSMZ, Braunschweig, Germany). Cells were maintained in Dulbecco’s modified Eagle’s medium (DMEM) and HAM’s F12 (1:1, *v*/*v*, Lonza, Basel, Switzerland) supplemented with 15% (*v*/*v*) heat-inactivated fetal bovine serum (FBS, Lonza, Basel, Switzerland) in T75 cell culture flasks at 95% H_2_O, 37 °C, and 5% CO_2_.

### 5.4. Fluorescence Microscopy

The localization of all generated receptor variants was monitored by live-cell fluorescence microscopy. 150.000 HEK293 cells in a total volume of 300 µL were seeded into 8-well µ-slides (Ibidi, Gräfelfing, Germany) and cultured to a confluence of 70%. The cells were then transfected with 500 ng of the respective receptor construct using Lipofectamine2000 (Invitrogen, Waltham, MA, USA) according to the manufacturer’s protocol. On the following day, the medium was removed and replaced with Opti-MEM (Thermo Fisher Scientific, Waltham, MA, USA) containing 2.5 µg/mL Hoechst 33342 nuclear stain (Thermo Fisher Scientific, Waltham, MA, USA). After incubation for 10 min_,_ the cells were subsequently monitored using an Axio Observer Z1 microscopic setup with ApoTome.2 (Zeiss, Oberkochen, Germany: 63×/1.4 oil objective, filter settings (ex/em): YFP 500 nm (20)/535 nm (30), DAPI 365 nm (20)/420 nm (30)). Identical acquisition times and image processing were applied for wild-type and receptor mutants.

### 5.5. cAMP Reporter Gene Assay

The activation of the G_s_ and G_i/o_ pathways was monitored by cAMP reporter gene assays. HEK293 cells were seeded into 6-well plates. At a confluence of 70%, the cells were co-transfected (MetafectenePro, Biontex, München, Germany) with 2 µg of the respective receptor construct and 2 µg of the pGL4.29 [CRE/Luc2P/Hygro] reporter gene (Promega, Madison, WI, USA, total 4 µg DNA in 1:1 ratio) according to the manufacturer’s protocol. The transfected cells were re-seeded into 384 well plates at a density of 15000 cells/well. After 24 h, the medium was replaced by 15 µL of a stimulation solution consisting of the respective peptides in unsupplemented DMEM, adding 2 µM Forskolin for G_i_-coupled receptors (Y_1_R, Y_2_R, MOR). As a negative control, cells were treated with DMEM, while for the positive control, cells were stimulated with DMEM containing 2 µM/10 µM Forskolin (G_i_/G_s_). All simulations were performed in technical triplicate. After 3 h of incubation, the cells were lysed with 15 µL of OneGlo solution (Promega, Madison, WI, USA). Luminescence values were measured after 5 min in a Spark plate reader (Tecan, Männedorf, Switzerland, t_integration_ = 500 ms). Data were normalized to the minimal (negative control) and maximal luminescence. The means of the independent experiments were pooled and fit to a three-parameter-based agonist (log) vs. response non-linear regression (GraphPad Prism 5, San Diego, CA, USA).

### 5.6. Inositol Phosphate Accumulation Assay

The activation of the G_q_ pathway was monitored by a commercial homogenous time-resolved fluorescence (HTRF)-based inositol phosphate (IP_1_) accumulation assay in transiently transfected HEK293 cells as described recently [70]. Briefly, HEK293 were co-transfected with 3.2 µg of the respective receptor construct and either 0.8 µg of an empty pcDNA3.1 vector (natively G_q_-coupled receptors) or 0.8 µg of a Giq4Δmyr-chimera (G_i_-coupled receptors) [34] using MetafectenePro (Biontex, München, Germany) according to the manufacturer’s protocol, and subsequently seeded into 384-well plates at a density of 20000 cells/well. On the next day, the medium was removed and replaced by 15 µL of a stimulation solution containing the respective peptide dilution in Hank’s balanced salt solution (HBSS) and 20 mM LiCl. For the detection of the inositol monophosphate, specifically labeled antibodies of the IP-One Gq kit (Cisbio, PerkinElmer, Waltham, MA, USA) were used according to the manufacturer’s protocol. The HTRF signal was read out using a Spark plate reader (Tecan, Männedorf, Switzerland, filter settings (ex/em): 320/620 nm (donor)–320/665nm (acceptor)). The measured values were normalized to the wild-type control (minimum/maximum response). The normalized means of the independent experiments were pooled and fit to a three-parameter-based agonist (log) vs. response non-linear regression (GraphPad Prism 5, San Diego, CA, USA).

### 5.7. Arrestin Recruitment Assay

Arrestin recruitment to the receptor variants was monitored by a BRET-based approach. HEK293 cells were seeded into 6-well plates. At a confluence of 70%, the cells were co-transfected (MetafectenePro, Biontex, München, Germany) according to the manufacturer’s protocol. A total of 4000 ng DNA was transfected per well. In the first set of experiments, we performed receptor titration experiments to determine the receptor-to-arrestin-ratio that ensures saturation of the luminescence donor (Nluc-arr3) by the fluorescence acceptor (receptor-eYFP) and hence, results in reproducible measurement window for ligand concentration-response curves. Accordingly, we chose to transfect 30 ng of a modified Nluc-tagged bovine arr-3 construct [71], 3900 ng receptor construct, and 70 ng of an empty pcDNA3.1 vector. After the transfection, 150,000 cells/well were re-seeded into solid white 96-well plates in technical triplicate using a phenol red-free culture medium. For the assay, the medium was replaced by a 100 µL of BRET buffer (HBSS + 20 mM HEPES, pH 7.4 50 µL of a coelenterazine H solution (Nanolight, Prolume, Pinetop, AZ, USA) 16.8 µM in BRET buffer) were added, and the plate was incubated for 5 min at 37 °C. 50 µL of the agonistic peptide solution were added (varying peptide concentrations between 0.01 nM to 10 µM; final volume in all conditions 200 µL). Fluorescence and luminescence values were measured well-wise at three time points (5, 10, and 15 min post-stimulation) using a Spark plate reader (Tecan, Männedorf, Switzerland, filter settings: 400–470 nm (luminescence); 535–650nm (fluorescence)). Raw BRET values were calculated well-wise by dividing the fluorescence values by the detected luminescence values. netBRET values were then calculated by subtracting the BRET values of the buffer control. The corrected values were either normalized to the respective wild-type receptor or the means were directly pooled and fit a three-parameter-based agonist (log) vs. response non-linear regression (GraphPad Prism 5, San Diego, CA, USA).

### 5.8. NanoBRET Ligand Binding Assay

BRET-based ligand binding assays were performed using membrane preparations of transiently transfected HEK293 cells expressing Nluc-Y_1/2_R-eYFP receptors as described recently [24]. Briefly, 0.03 µg total protein per well was suspended in ice-cold HBSS containing 20 mM HEPES (pH 7.4), Pefabloc, and 0.1% bovine serum albumin (BSA), corresponding to a total luminescence of 500.000–1.000.000 RLU. The assay was performed in solid black 96 WP with a total assay volume of 100 µL. The TAMRA-labeled peptides were prepared as 10× stock in HBSS containing 20 mM HBSS/HEPES (pH 7.4) and 0.1% BSA, added to the membranes and incubated for 10 min at room temperature with gentle agitation (total binding). To determine unspecific binding, 100-fold excess of unlabeled NPY or specific receptor antagonist BIBP3226 (Y_1_R; [72]) or JNJ-31020028 (Y_2_R; [73]) was added.

10 µL of coelenterazine H (42 µM) in HBSS/HEPES (pH 7.4) were added to each well, and BRET was measured using a Spark plate reader (Tecan, Männedorf, Switzerland, well-wise mode, filter settings: 430–470 nm (luminescence); 550–700 nm (fluorescence)). Raw BRET values were calculated well-wise by dividing the fluorescence values by the detected luminescence values. NetBRET values were calculated by subtracting the BRET values of buffer controls. Specific binding was determined by correcting the total binding for unspecific binding. The corrected values were fit in a biphasic non-linear regression and plotted as a function of the concentration of the labeled peptide (GraphPad Prism 5, San Diego, CA, USA).

### 5.9. Cell Surface ELISA

Membrane expression of selected receptor variants was confirmed by cell surface ELISA. HEK293 were seeded in 6 WP and transiently transfected with 3 µg DNA using MetafectenePro (Biontex, München, Germany) according to the manufacturer’s protocol. The cells were re-seeded into poly-D-lysine-coated transparent 96-well plates at a density of 150,000 cells/well and cultivated overnight in complete medium, followed by 30 min serum-deprivation using Opti-MEM (Thermo Fisher Scientific, Waltham, MA, USA). The cells were subsequently fixated with 50 µL PFA (2% in phosphate-buffered saline (PBS)) on ice for 60 min and washed three times with 150 µL PBS. Next, unspecific binding was blocked with full medium (surface receptors) or full medium + 0.5% Triton-X 100 (permeabilization to determine total receptors) for 60 min at room temperature. The cells were rinsed once with 150 µL PBS per well. Primary antibodies were diluted in DMEM + HAM’s F12 (1:1, *v*/*v*, + 7.5% FBS *v*/*v*) as follows: anti-AT_1_R (ab124734, rabbit, Abcam, Cambridge, UK) 1:1000, anti-MOR (SAB4502048, rabbit, Sigma-Aldrich, St. Louis, MO, USA) 1:1000, anti-GLP_1_R (sc-390774, mouse, Santa Cruz Biotechnology, Dallas, TX, USA) 1:60. Dilutions of the respective primary antibodies were incubated for 120 min at room temperature. A negative/specificity control was included for every receptor and incubated with DMEM + HAM’s F12 (1:1, *v*/*v*, + 7.5% FBS *v*/*v*) without a primary antibody. The cells were then washed three times with 150 µL PBS per well. Horseradish peroxidase- (HRP) coupled Anti-Rabbit IgG H&L (AT_1_R, MOR, ab205718, goat, Abcam, Cambridge, UK, 1:10000) and m-IgGκ BP (GLP_1_R, sc-516102, Santa Cruz Biotechnology, Dallas, TX, USA, 1:1000) antibodies were diluted in DMEM + HAM’s F12 (1:1, *v*/*v*, + 7.5% FBS *v*/*v*) and incubated for 120 min at room temperature. The cells were washed four times with 150 µL PBS per well and subsequently incubated with 100 µL of 3,3′,5,5′-tetramethylbenzidine (TMB) for 3 min. The reaction was stopped with 100 µL of 0.25 M HCl to obtain a stable yellow color, and the absorption at 450 nm was measured in an Infinite M200 Plate reader (Tecan, Männedorf, Switzerland). Absorption values were corrected by subtracting the mean of the negative controls of the respective receptor. The resulting values of the non-permeated samples (membrane expression) were divided by the values of the permeated samples (total expression). The assays were conducted 2–7 times independently in technical triplicate. The means of the corrected values from the individual assays were pooled and displayed as fractions of the respective total expression.

### 5.10. In Vitro Sample Preparation of Y2R-A202^45.49^C, Δ6Cys in Small DMPC/DHPC Bicelles for CW-EPR

Sample preparation of the cysteine deficient Y_2_R_A202^45.49^C,Δ6Cys variant in small 1,2-dimyristol-sn-glycerol-3-phosphocholine (DMPC)/1,2-diheptanoyl-sn-glycero-3-phosphocholine (DHPC) bicelles was performed as described previously [39,74]. Briefly, the cDNA of Y_2_R_A202^45.49^C-Δ6Cys-polyHis was introduced into a pET41b vector system, and proteins were expressed as inclusion bodies during a fed-batch fermentation in *Escherichia coli* NiCo21 cells, followed by solubilization in a sodium phosphate buffer supplemented with 15 mM SDS and 50 mM DTT. The receptor was purified by metal affinity chromatography (NiNTA). Before in vitro folding and nitroxide spin labeling, the sample was diluted to 0.5 mg/mL and treated with 5 mM TCEP to fully reduce the three remaining cysteines of the receptor and thus prevent unspecific disulfide bridges or oligomer formation. TCEP was then removed by several dialysis steps against 25 mM degassed TRIS/HCl containing 15 mM SDS (pH 7). Next, the SDS concentration was decreased to 1 mM while introducing a redox-shuffling system (1 mM GSH and 0.5 mM GSSG) to facilitate the formation of the conserved disulfide bond. Subsequently, the receptor was reconstituted into small DMPC/DHPC bicelles (q < 0.25) using a molar ratio of 1: 600: 2400 of receptor: DMPC: DHPC. Spin-labeling of the single remaining free cysteine at position A202^45.49^C in the extracellular loop 2 with 3-(2-iodacetamido)-proxyl (IAP) was achieved by adding three-times 10-fold molar excess to the receptor sample (incubation 2 × 2 h at room temperature and 1× overnight at 4 °C). To fully remove unbound spin-label from the sample, the receptor was (partially) unfolded and refolded a time using the above protocol excluding the reducing agent and redox-shuffling system. Spin concentration produced the final sample used for the EPR experiments containing ~30 µM P1 labeled receptor in 25 mM TRIS/HCl buffer, pH 7.

### 5.11. CPM Assay

Disulfide bridge formation and spin labeling were evaluated in a fluorescence-based assay system using a thiol-reactive fluorochrome N-[4(7-diethylamino-4-methyl-3-coumarinyl) phenyl]maleimide (CPM) as described previously [39]. A 4 mg/mL CPM stock solution was prepared in DMSO and further diluted 40-fold in 25 mM Tris/HCl buffer pH 7 to a concentration of 0.1 mg/mL. Simultaneously, a total amount of 5 µg receptor protein from each sample was re-suspended in 15 mM SDS containing 25 mM Tris/HCl buffer, pH 7, to a final volume of 360 µL. The receptor samples were then mixed with 30 µL of the prepared CPM solution and incubated in the dark for 30 min at room temperature. Fluorescence intensities were determined on a Spark Reader (Tecan, Männedorf, Switzerland) using an excitation wavelength of 387 nm, scanning emission wavelength from 450 to 500 nm, and an integration time of 0.5 s. All samples were scanned three times at 20 °C.

### 5.12. Fluorescence Polarization Assay

To determine the ligand binding capacity of the Y_2_R_A202^45.49^C in small bicelles and the IAP-labeled variant, we performed a fluorescence polarization assay as described previously [74]. Therefore, various concentrations of the receptor constructs were incubated with 50 nM (K^18^-TAMRA)-NPY for 1 h at room temperature and transferred as triplicates in a 96-well plate (Corning, non-binding surface, # CLS3881, Corning, NY, USA). Fluorescence polarization was measured using the Spark Microplate Reader (Tecan, Männedorf, Switzerland) with linearly polarized light, an excitation wavelength of 549 nm, an emission wavelength range of 574 to 578 nm, and a 90° detection angle at 20 °C. A sigmoidal dose-response curve was used to fit the data with the ‘OriginPro,2019’ software.

### 5.13. Continuous-Wave (CW)-EPR Measurements

The following sample set was prepared for CW-EPR measurements: (1) a control sample of free IAP (100 µM) with empty bicelles in Tris/HCl buffer pH 7 and (2) Y_2_R_A202^45.49^C,6Cys-IAP (A202^45.49^-P1, c = 40 µM) in small bicelles. Next, titration experiments with increasing concentrations of the reduction reagent TCEP were performed. A 50 mM TCEP stock solution in 25 mM TRIS/HCl buffer (pH 7) was prepared, and aliquots of A202^45.49^-P1 were then treated for 45 min at room temperature with TCEP in a range of 0.001 mM to 5 mM. All samples were added to ‘50 µL-Blaubrand’ borosilicate capillaries (I.D. 0.85 mm, O.D. 1 mm, ref.-number: 708733, Brand GmbH, Wertheim, 97877 Germany, Germany).

X-Band (~9.6 GHz) room temperature CW-EPR spectra were obtained on a Bruker EMXmicro spectrometer (Bruker, Karlsruhe, Germany). Measurements were performed using the high-sensitivity dielectric resonator (ER4123D) at 20 db attenuation (2 mW), 100 kHz modulation frequency, and modulation amplitude of 0.3 mT. The final spectra represent an average of 150 scans, using a scan width of 12 mT at a sampling rate of 0.8 mT/s.

EPR spectra were simulated using the software package MultiComponent v.1034 (developed by the laboratory of Wayne Hubbell at UCLA, available under https://www.biochemistry.ucla.edu/Faculty/Hubbell/software.html) (accessed on 9 January 2023). The default values for magnetic (g) and hyperfine (A) tensors were used (g_xx_ = 2.0078, g_yy_ = 2.0058, g_zz_ = 2.0023, A_xx_ 5.7 G and A_yy_ = 6 G) and first validated in the simulation of free IAP (P1) in the empty bicelles spectrum. The isotropic simulation yielded the rotational correlation time of τ_c_ = 45 ps and the hyperfine splitting tensor A_zz_ of 36.12 G. In the next step, we simulated the spectra of Y_2_R-A202C^45.49^-P1 treated with 5 mM TCEP applying a three-component system with variable R1, A_zz_, phase, and B_0_, plus amplitudes (scales). The most relevant fitting parameters are for the fast component A_zz_ = 36.1 G, t_c_ = 0.282 ns; for the intermediate component A_zz_ = 37.71 G, t_c_ = 2.62 ns and for the immobilized component A_zz_ = 34.76 G, t_c_ = 10.7 ns. Including ordering potentials, motional anisotropy, or line broadening in either of the components led to only negligible improvements in the fit. To fit the spectra recorded at variable TCEP concentrations, we used the same motional model with variable component amplitudes. These amplitudes and their corresponding errors were normalized to unity and plotted vs. TCEP concentration using the data analysis software ‘OriginPro2019’. The equivalence point of the titration curve was calculated based on the fitting formula:

$$F\left(x\right)=A1+\frac{A2-A1}{1+10^{\left({Log}_{x}0-x\right)p}}$$

with *A*1 = bottom asymptote, *A*2 = top asymptote and *p* = Hill slope.

## Data Availability

Data can be obtained from the authors upon reasonable request.
